# Cognitive Impairment and Decline in Physical Function Among Older Mexican Americans Over 20 Years

**DOI:** 10.1093/geroni/igaa057.3303

**Published:** 2020-12-16

**Authors:** Amy Givan, Soham Al Snih

**Affiliations:** The University of Texas Medical Branch at Galveston, Galveston, Texas, United States

## Abstract

The aim of this study was to examine cognitive function as a predictor of physical function decline over a 20-year follow-up period among older Mexican Americans who were non-disabled and were able to complete the Short Physical Performance Battery (SPPB) at baseline. The sample consisted of 2,232 Hispanics 65 years and older from the Hispanic Established Population for the Epidemiological Study of the Elderly. Measures included socio-demographics, self-reported medical conditions, body mass index (BMI), disability, depressive symptoms, limitations in activities of daily living, Mini-Mental State Examination (MMSE), and SPPB. General linear mixed models were used to estimate changes in SPPB over time as a function of MMSE. At baseline, 11% of the participants had cognitive impairment (MMSE < 21) and the average SPPB score for those with and without cognitive impairment was 7.16 + 2.75 and 7.81 + 2.36, respectively. Mixed model analysis showed that those with cognitive impairment (MMSE < 21) experienced a decline in the SPPB of 0.34 points per year (Standard Error = 0.09, p-value = 0.0002) after controlling for all covariates. Other significant predictor factors of decline in the SPPB were older age, depressive symptoms, diabetes, any assistance with activities of daily living, having had a hip fracture, and high BMI. Cognitive impairment predicted decline in physical function among older Mexican Americans who were non-disabled at baseline. These findings underscore the need of developing interventions to maintain cognitive and physical function to delay or prevent disability in this underserved population with high rates of disability.

